# Dispositional optimism and business recovery during a pandemic

**DOI:** 10.1371/journal.pone.0269707

**Published:** 2022-06-09

**Authors:** Mario Amore, Orsola Garofalo, Victor Martin-Sanchez

**Affiliations:** 1 Department of Management and Technology, Bocconi University, Milan, Italy; 2 Centre for Economic Policy Research (CEPR), London, United Kingdom; 3 Department of Strategy and Innovation, Copenhagen Business School, Frederiksberg, Denmark; 4 Department of Business and Management, University of Southern Denmark, Odense, Denmark; Universiti Pertahanan Nasional Malaysia, MALAYSIA

## Abstract

A large literature at the crossroads of biology and cognitive psychology has shown that individuals hold generally positive expectations about future events. Despite this evidence, to date it remains unclear whether optimism has positive or negative implications for entrepreneurial activities. We examine this question in the context of the Covid-19 pandemic, which provides a unique way to study the role of optimism on the (in)ability of firms to overcome exogenous shocks. Using a large-scale longitudinal survey covering 1,632 UK firms, we find that entrepreneurs who score high on optimism were more likely to innovate and make organizational changes to their firms during the Covid-19 outbreak. Moreover, optimistic entrepreneurs experienced higher revenue growth during the pandemic. Collectively, our study sheds light on one of the psychological factors explaining why some firms can prosper and some others struggle in the wake of an external shock.

## Introduction

Several existing studies indicate that humans are generally optimistic, i.e., they display a tendency to expect positive events in the future even when the available evidence does not support such expectation [1–3]. Dispositional optimism has been shown to play a key role in a wide range of human activities related to work, social relations, and health [4]. Within this literature, several studies have further documented a positive association between optimism and individual health [5]: optimistic individuals tend to experience a lower mortality risk [6] as a result of their higher propensity to take proactive steps to protect health [5] and their better emotional response to adversities, which in turn reduces the physiological strain over time [4]. In particular, optimism has been shown to be negatively associated with the risk of stroke [7], rehospitalization following coronary artery bypass surgery [8], and chronic diseases of aging and premature mortality [9]. The meta-analysis in [10] confirms that optimism is a significant predictor of positive health outcomes.

An important, yet unaddressed, question concerns whether optimism can matter not only for individual survival but also for the survival of entrepreneurial firms in the wake of negative events. This question is relevant considering the notoriously high mortality rate of new ventures, which has triggered an important debate about why some firms are better equipped than others to survive [11, 12].

Psychology plays a key role in entrepreneurial decision-making. Several works in this realm have shown that entrepreneurs tend to display a particularly high level of optimism, which translates into a higher confidence in their ability to succeed [13]. However, as for any personal trait, optimism varies across individuals due to genetic and environmental factors [4]. Focusing on dispositional optimism is important because it may underlie variations in entrepreneurial actions, which in turn map into business outcomes [13–15]. The mechanism linking optimism and business outcomes is that entrepreneurs act on their confidence and personal beliefs when deciding upon the allocation and use of resources [16, 17]. Despite a consensus on the view that optimism influences entrepreneurs’ decision-making, it remains unclear whether this influence is positive or negative for entrepreneurial firms. We examine this question by focusing on the Covid-19 pandemic, which created many unprecedented business challenges ranging from value-chain disruptions and organizational setbacks due to employee illness to problems in managing customer relationships in ways consistent with lockdown policies. While these challenges threatened the survival of many entrepreneurial firms [18], they also brought about opportunities emerging from changes in households’ daily routines and new customer needs. Coping with these challenges and turning them into opportunities requires entrepreneurs to pivot their current ways of doing business along many dimensions spanning from product innovation to organizational and supply-chain adjustments. Indeed, innovation is one of the key strategic responses that firms may enact amidst a crisis [19, 20].

On the one hand, optimism may have raised the ability of entrepreneurs to imagine pathways to overcome pandemic-related challenges and behave more proactively. This view stems from the idea that optimism is positively associated with a successful adaptation to stressful events [21, 22] and with the ability to undertake organizational changes [23]. On the other hand, optimism may have created an illusory sense of control, exposing entrepreneurs to heuristics and biases, and leading them to errors in judgment [24]. These features may be especially problematic in times of crisis, which make resources more scarcely available and the cost of mistakes potentially larger. Did dispositional optimism improve or worsen the entrepreneurial response to the pandemic shock?

To answer this question, we employ a large multi-wave survey covering entrepreneurs based in the United Kingdom (UK). We start by showing a large negative impact of Covid-19 on our sample firms: one third of them were completely closed during the spring lockdown, and 72% of them reported a revenue drop (which amounts to 37%, on average). Yet, several entrepreneurs took actions to cope with Covid-19: around 30% of them tried to innovate their products or processes, whereas 25% of them changed their organizational structure. The key finding of our study is that dispositional optimism explains large variations in the likelihood of such entrepreneurial actions during the pandemic: optimistic entrepreneurs were more proactive in making organizational changes and innovate during the Covid-19 outbreak. In parallel, optimistic entrepreneurs have more positive beliefs regarding the time needed for their business to fully recover from the crisis, and a higher likelihood to experience a revenue increase in the future (even controlling for the size of revenue drop). Finally, optimistic entrepreneurs hold more positive expectations about macroeconomic conditions. Those actions and beliefs translate into superior business outcomes: our data reveal a positive association between dispositional optimism and growth during the pandemic period.

Collectively, our results suggest that dispositional optimism is significantly associated with effective entrepreneurial actions in the wake of the Covid-19 pandemic. Our work contributes to research on the implications of Covid-19 for entrepreneurship and management [18, 25] and, more generally, on the drivers of business resilience during hard times [26–29]. It also complements existing works on how individual beliefs [30] and economic expectations [31, 32] evolved during the pandemic. Finally, we expand the large literature on how optimism affects business outcomes by leveraging on a new context such as the Covid-19 pandemic, which prompted many entrepreneurs to engage in business actions functional to overcome the pandemic period. While, as said, the association between optimism and performance is still debated in the literature, our results point to a beneficial role of optimistic tendencies during a time of crisis.

## Materials and methods

### Context

Covid-19 has proven to be one of the most dreadful viruses of the recent human history. In the UK, the virus experienced a rapid diffusion starting from late February (i.e. when the first case was identified): the daily number of infections reached its maximum on April 9^th^ (with 5,450 cases), whereas the daily number of deaths peaked on April 22^nd^ (with 1,224 deaths). Data are drawn from: https://covid19.who.int/region/euro/country/gb. The surge of Covid-19 in the UK led to a four-month lockdown (from March to June 2020), which constrained the activities of 5.8 million among entrepreneurs, small and medium-sized enterprises and self-employed [33]. Importantly, contrary to the second outbreak that affected many countries in the fall of 2020, the spring outbreak was unprecedented and entirely unexpected. Thus, it provides a useful context to analyze the readiness of entrepreneurial actions to the pandemic.

### Survey design

All participants in this study gave informed consent, and ethical approval was provided in written form and before data collection started by the Research Ethics Office of King’s College London, the institution where Victor Martin-Sanchez was based at the time of starting this project.

Survey methods have been widely used to understand the impact of Covid-19 on the entrepreneurship landscape; e.g. [34]. We sent our first survey through Prolific Academic at the beginning of June 2020, during which the Covid-19 shock has, at least in part, already been incorporated into revenues, and entrepreneurs had time to implement business actions to face the pandemic. The survey was sent to 2,000 entrepreneurs whose business was based in the UK. To encourage participation in the survey, we provided a participation fee of 5£ per hour, as well as three lottery prizes of 300£, 150£ and 150£. Additionally, there was a set of incentivized questions where participants could get an extra 0.30£ by: (1) providing the range-estimate of the number of Covid-19 contagion cases in the UK at end of July; (2) providing the position of the UK in the ranking of the countries with most contagion cases; and (3) being randomly selected in one of the Holt-Laury questions (explained later). The survey contains a total of 30 questions regarding entrepreneurs’ demographic and business characteristics (before and during the spread of Covid-19), as well as questions regarding the specific actions taken during the pandemic period, and expectations about the future diffusion of Covid-19. We received 1,632 usable responses, which amount to an 82% response rate. The final sample satisfies the following criteria: (1) the respondents are currently business owners; and (2) they reside in the UK.

Dispositional optimism, i.e. the key explanatory variable in our study, can be thought as a general expectation that more good things, rather than bad, will happen in the future [3, 35]. To measure the extent to which individuals expect that good (resp. bad) things will be plentiful, we use the revised Life Orientation Test (LOT-R), developed in [35] and widely employed in business research [14, 15, 36, 37]. The LOT-R has ten items: three (questions 1, 4, and 10) measure optimism, another three (questions 3, 7, and 9) measure pessimism, and four items (questions 2, 5, 6, and 8) serve as “fillers”. Respondents rate each item on a 5-point scale: “Strongly disagree” (0 points), “Disagree” (1 point), “Neutral” (2 points), “Agree” (3 points), or “Strongly agree” (4 points). The final measure of dispositional optimism is computed by summing the scores of questions related to optimism and pessimism, where the latter are reverse-coded. It ranges from 0 to 24, and higher values indicate a higher level of optimism.

Next, we follow [38] to assess individuals’ risk preferences (which can matter too in responding to the pandemic and hence confound the effect of optimism). In the Holt and Laury’s framework, every player is given a list of ten rows of paired gambles and for each gamble he/she must indicate a preference by choosing either Option A or Option B. The payoffs of gambles in the two options are constant but they differ in the probability of each payoff: for the first pair of gambles there is a 10% chance of receiving the high payoff for either option, while for the last pair of gambles there is a 100% probability of receiving the high payoff. What usually occurs is that Option A is chosen for the first decision and then, at some point before the last decision row, the respondent switches to Option B. The switching point measures individual risk preferences: the higher the point, the greater the individual’s risk aversion.

To shed light on the effectiveness of entrepreneurial actions implemented during the spring of 2020, we conducted another survey in May 2021 in which we asked the same entrepreneurs questions about their firms’ revenues and employees, together with a few other questions (including optimism, described above). Of the 1,632 respondents to the survey sent in June 2020, we were able to get usable responses from 996 entrepreneurs.

#### Sample characteristics

Table 1 shows the summary statistics for the main individual and business-related variables (taken from the first survey wave). As shown, the entrepreneurs in our sample are on average 40 years old, 64% of them are women, and 19% hold an advanced degree (Master or PhD). The average level of optimism is equal to 14, which is at par with the value found in Scheier et al. (1994). Examining business characteristics, we find that the average firm is 7 years old and has 1.8 full-time employees as of December 2019. The subsequent rows show that Covid-19 caused a significant economic downturn: one third of entrepreneurs reported that their business was entirely closed during the spring lockdown, and 72% of them reported a drop in revenues. The average change in revenues in January-April 2020 relative to the same period in 2019 was -37%. While we will use revenue change in the analysis, our data also contain information on employees. The average change in the number of total employees was -9%.

**Table 1:** Summary statistics.

	Observations	Average	Median	s.d.
Dispositional optimism	1,632	14.012	14	5.038
Advanced education	1,632	0.193	0	0.395
Female	1,632	0.643	1	0.479
Age	1,632	40.583	39	11.776
Risk aversion	1,632	5.928	6	3.103
Founding year	1,632	2012.79	2015	8.299
Employees pre Covid-19	1,632	1.779	0	8.454
Closed during Covid-19	1,632	0.319	0	0.466
Revenue drop indicator	1,632	0.724	0	0.447
Revenue change during Covid-19	1,632	−37.785	−40	52.998

Note: Dispositional optimism is a continuous measure computed as the sum of the entrepreneurs’ answers (on a Likert-based scale: 0 = strongly disagree, 1 = disagree, 2 = neutral, 3 = agree, 4 = strongly agree) to a number of questions contained in the revised LOT developed by Scheier et al. (1994). Female is a dummy for women, zero otherwise. Age measures the entrepreneur’s age (as of 2020). Advanced education is a dummy equal to one if the entrepreneurs holds a Master or PhD degree, zero otherwise. Risk aversion is a continuous variable computed using the test in [38]. Founding year is the year when the business was founded. Employees pre Covid-19 is the number of full-time employees as of December 2019. Revenue drop indicator is a dummy equal to one if the business has reported a decline in revenues in January-April 2020 as compared to the same period in 2019. Revenue change during Covid-19 is the percentage change in revenues in January-April 2020 as compared to the same period in 2019.

**Fig 1:**
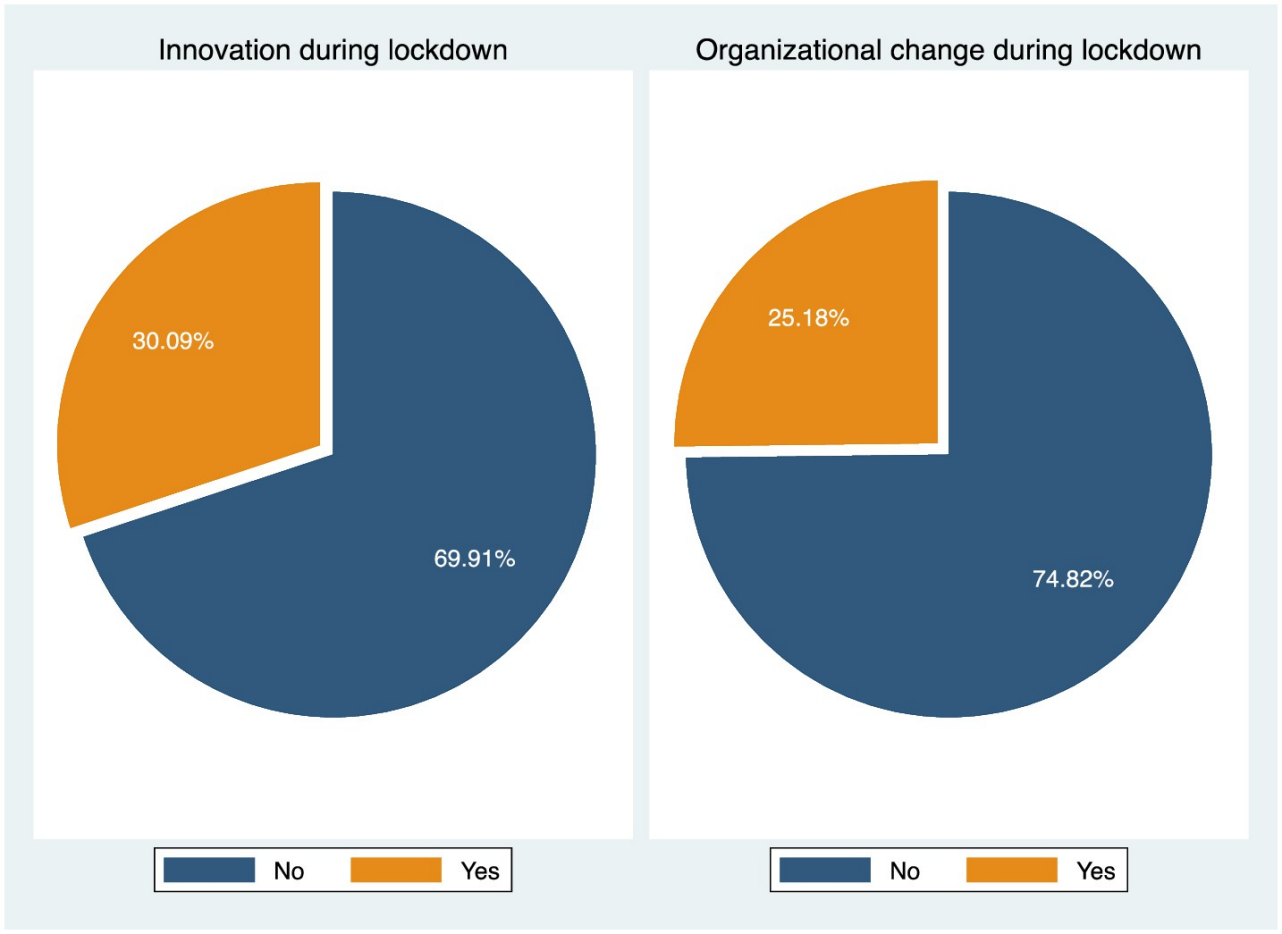
Entrepreneurial actions during Covid-19. Note: The figure indicates the fraction of businesses which report to have introduced product or process innovation, and organizational changes during January-April 2020.

Focusing on the actions undertaken to face the Covid-19 pandemic, Fig 1 shows that almost one fourth of the entrepreneurs in our sample made organizational changes to their business, whereas one third of them in introduced some form of product or process innovation.

Our survey contains two questions where we asked entrepreneurs to provide a textual description of innovation and organizational adjustments. Parsing this information, we found that the three most frequently cited types of innovation are in the area of digital systems (66%), product or process expansion (13%), and delivery systems (8%). The three most frequently cited types of organizational change are about workplace adjustments, e.g. flexible work schedule, remote-working etc. (33%), job retention schemes (20%), and business model adjustments (12%). Our survey also contains a question regarding whether the entrepreneurs have applied to any governmental support scheme; 35% of them have done so (with no systematic differences by the level of dispositional optimism).

**Fig 2:**
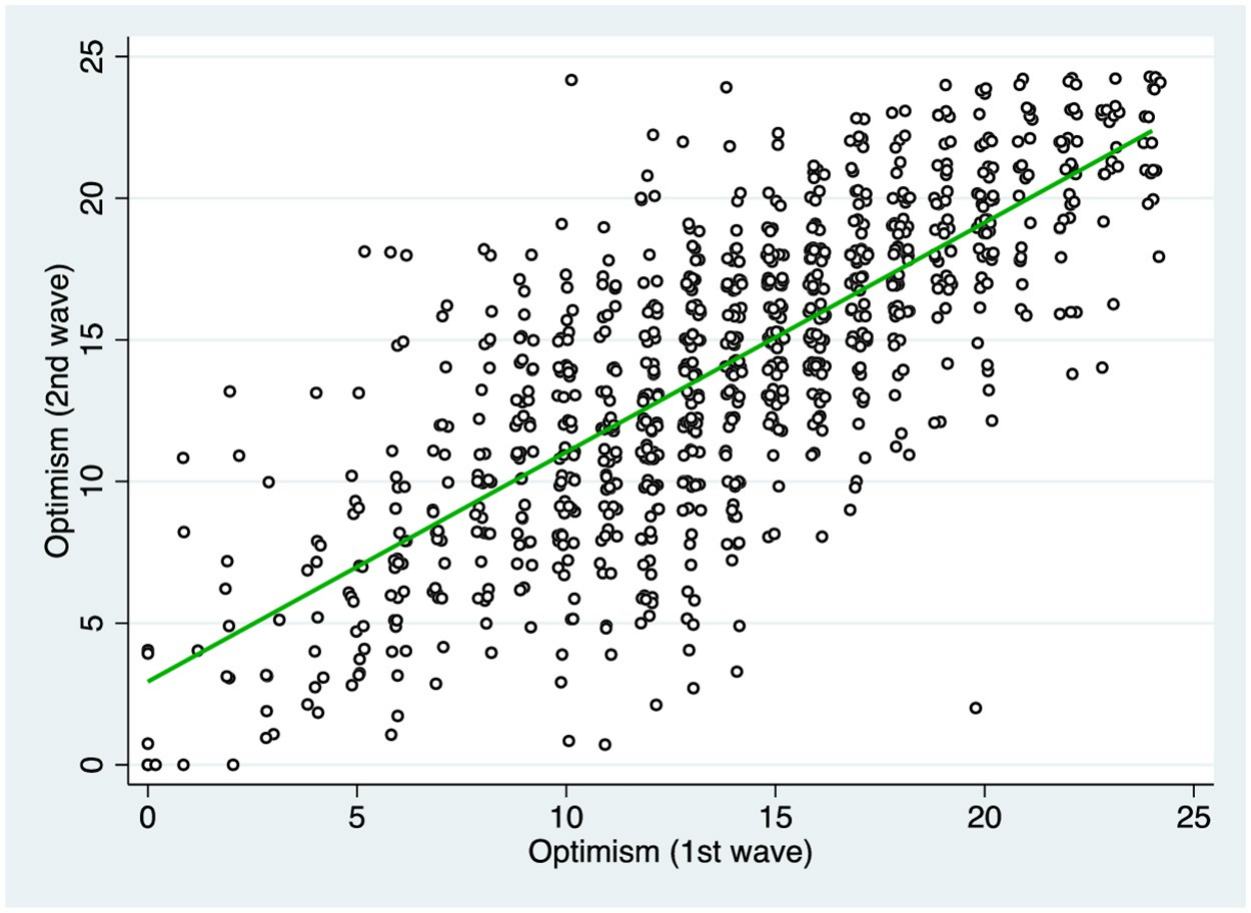
Stability of optimism over time. Note: The figure illustrates the relationship between optimism in the first survey wave (June 2020) and the second wave (May 2021). The dots represent the observations whereas the green line is the linear fit.

Our measure of optimism used so far is based on individual beliefs during the uptake of the virus. This raises the question of whether there was a reverse-causality mechanism whereby better business outcomes may have made entrepreneurs more optimistic. In addressing this point, it is useful to keep in mind that optimism is a highly stable trait [5], which features small within-subject variations even in the wake of drastic life events [39]. As anticipated, our second survey contains a set of questions which—for a given individual—enable us to compute dispositional optimism at two different points in time (i.e. June 2020 and May 2021). Consistent with the notion of stability of optimism, the correlation between these two measures is 0.77. Fig 2 shows the positive association (and linear fit) between optimism over time.

## Results

### Entrepreneurial actions during the spring lockdown

As we discussed, several works in the literature suggest that optimistic entrepreneurs tend to engage in potentially wasteful actions; others suggest that optimistic entrepreneurs are better positioned to make proactive actions to improve performance in the wake of aversities. Theoretically, these mechanisms can have opposite effects on the ability to overcome a pandemic. We move to the data to understand the sign of this relationship.

**Fig 3:**
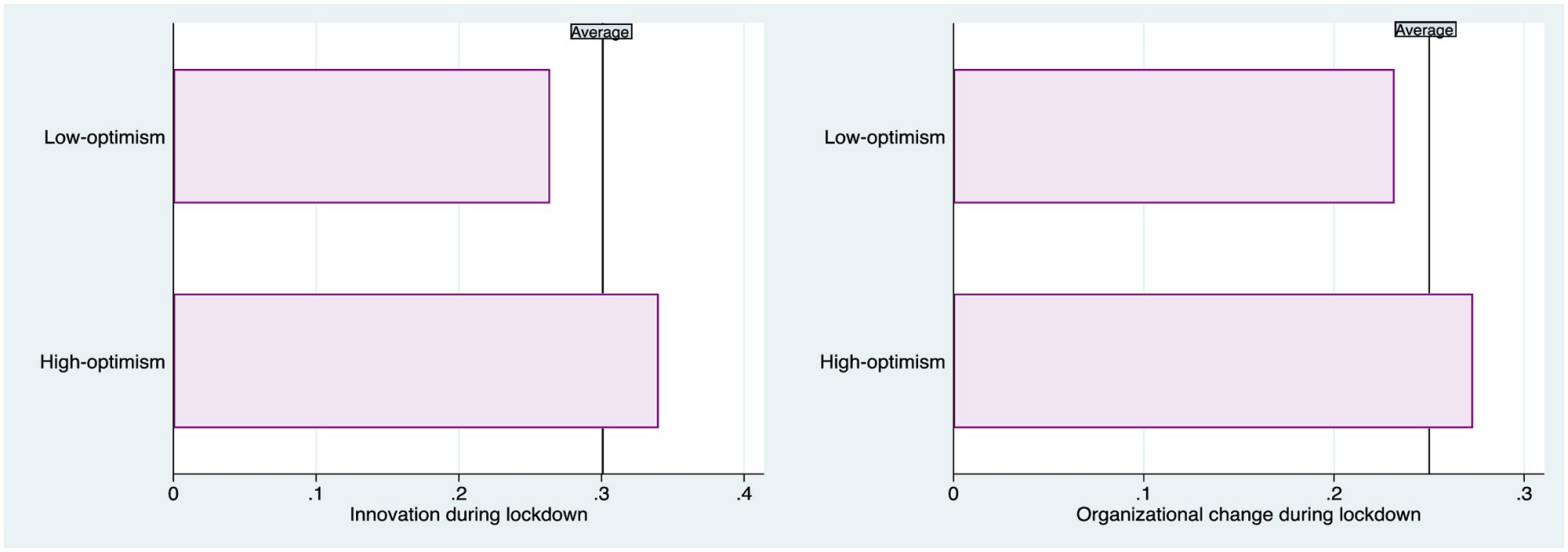
Entrepreneurial actions during Covid-19 –by optimism level. Note: The figure on the left (right) indicates the fraction of businesses which report to have made any innovation (organizational change) during January-April 2020, separately for entrepreneurs below and above the median threshold of optimism. The vertical line denotes the average fraction.

Our first piece of evidence concerns the association between optimism and organizational change or innovation during the pandemic. We probe into this issue in Fig 3, which shows that high-optimism entrepreneurs were much more likely to both innovate (in terms of products or processes) and make organizational changes to their business in the period from January to April 2020.

We confirm these results in Tables 2 and 3, where we estimate a set of linear probability models in which the dependent variables are, respectively, a dummy identifying instances of organizational change (Table 2) or innovation (Table 3) made during the Covid-19 pandemic. In these regressions, the key explanatory variable is dispositional optimism. Standard errors are adjusted for heteroskedasticity.

**Table 2:** Optimism and organizational change during the Covid-19 pandemic.

Dependent variable: Organizational change					
	(1)	(2)	(3)	(4)	(5)
Dispositional optimism	0.0056	0.0062	0.0056	0.0057	0.0053
	(0.007)	(0.003)	(0.007)	(0.006)	(0.009)
Revenue change during Covid-19		−0.0007	−0.0006	−0.0007	−0.0008
		(0.000)	(0.000)	(0.000)	(0.000)
Advanced education				0.0745	0.0670
				(0.010)	(0.020)
Female				−0.0216	−0.0137
				(0.357)	(0.550)
Age				−0.0027	−0.0032
				(0.003)	(0.002)
Risk aversion				−0.0078	−0.0068
				(0.023)	(0.045)
Founding year					−0.0017
					(0.234)
Closed during Covid-19					0.0501
					(0.040)
Employees pre Covid-19					0.0084
					(0.004)
Industry dummies	No	No	Yes	Yes	Yes
Observations	1,632	1,632	1,632	1,632	1,632

Note: The dependent variable is a dummy equal to one if the business has made any organizational change in January-April 2020, and zero otherwise. The main explanatory variable is the continuous measure of dispositional optimism. Depending on the specification, the regressions also include a host of individual and firm-level controls, and industry dummies (coefficients not shown for brevity). p-values are reported in parenthesis.

**Table 3:** Optimism and innovation during the Covid-19 pandemic.

Dependent variable: Innovation					
	(1)	(2)	(3)	(4)	(5)
Dispositional optimism	0.0073	0.0071	0.0073	0.0065	0.0065
	(0.001)	(0.001)	(0.001)	(0.003)	(0.003)
Revenue change during Covid-19		0.0001	0.0002	0.0001	−0.0003
		(0.547)	(0.397)	(0.509)	(0.245)
Advanced education				0.1058	0.1003
				(0.001)	(0.001)
Female				−0.0164	−0.0116
				(0.497)	(0.628)
Age				−0.0005	−0.0008
				(0.646)	(0.444)
Risk aversion				−0.0076	−0.0079
				(0.034)	(0.028)
Founding year					−0.0006
					(0.688)
Closed during Covid-19					0.1371
					(0.000)
Employees pre Covid-19					0.0030
					(0.001)
Industry dummies	No	No	Yes	Yes	Yes
Observations	1,632	1,632	1,632	1,632	1,632

Note: The dependent variable is a dummy equal to one if the business has made any product or process innovation in January-April 2020, and zero otherwise. The main explanatory variable is the continuous measure of dispositional optimism. Depending on the specification, the regressions also include a host of individual and firm-level controls, and industry dummies (coefficients not reported for brevity). p-values are reported in parenthesis.

As shown in Column (1), optimism is positively associated with the likelihood of organizational and innovation upgrades: the coefficients of dispositional optimism are all positive and their significance is consistently around the 1% level.

Our regressions include a large set of control variables at the individual and business level to ameliorate concerns of omitted factor bias. An important driver of the tendency to make changes to the business during the pandemic was its exposure to the Covid-19 shock: firms that were more severely affected by Covid-19 (e.g. because they operate in industries directly hit by the lockdown policies) may have featured stronger incentives to make organizational changes; in turn, the revenue change of a firm during the pandemic may have been associated with the entrepreneur’s optimism (e.g. because of a different sorting of optimists and pessimists across sectors). To account for this potential source of omitted factor bias, in Column (2) of Tables 2 and 3 we control for the percentage change in revenues during the pandemic (which in our descriptive table we reported to be around -37%). Additionally, in Column (3) we explicitly control for industry effects by means of a (16-grouping) set of industry dummies (see S1 File). Even keeping revenue change constant across entrepreneurs and estimating within-industry effects, dispositional optimism is positively associated with organizational change and innovation during the pandemic.

In the subsequent columns, we further control for a host of firm characteristics like the year when the business was founded, the number of employees before Covid-19, and a dummy equal to one whether the business was closed during the lockdown, as well as individual characteristics such as the entrepreneur’s age, his/her risk-aversion, gender, and educational attainment. The coefficients of these control variables indicate that more educated and risk-prone entrepreneurs were better equipped to react to Covid-19. That said, our main finding withstands the inclusion of these controls: in economic terms, the results in the most comprehensive specification (Column 5) indicate that a standard deviation increase in dispositional optimism is associated with a 2.5 times higher probability of organizational change, and 3 times higher probability of innovation.

### Entrepreneurial expectations

Expectations are key in entrepreneurial decision-making because they guide the selection of strategic reference points and organizational aspirations. In this section, we explore how optimism shaped entrepreneurs’ expectations in the period following the spring lockdown (i.e. from June 2020 onward). We organize this analysis around three dimensions: (1) business-related outcomes; (2) diffusion of the pandemic in the UK; (3) macroeconomic conditions.

We start by providing some descriptive evidence by using the answers to a survey question about the estimated period necessary to restore revenues to the pre Covid-19 level of December 2019. Inspecting the distribution of this variable, we find that 80% of entrepreneurs expect a full recovery within one year. However, this finding varies depending on the level of optimism: 57% of high-optimism entrepreneurs (i.e. above the median threshold) expect a recovery within 6 months, whereas the fraction is 6 percentage points lower for low-optimism entrepreneurs (see S1 File). Of course, the estimated recovery period depends on the size of the revenue drop and other business characteristics. Hence, to provide more compelling evidence, we estimate a regression in which the dependent variable is a continuous measure of the recovery period (in months), and the explanatory variables are entrepreneurs’ optimism together with the set of firm- and individual-level controls used so far. Results in Table 4 show that a standard deviation increase in optimism is associated with about a half-year faster expectation of revenue recovery. Importantly, this result is derived by controlling for the drop in revenues experienced during the spring lockdown. In Columns (2) and (3) of the table, we further show that optimism is positively associated with the expectation that revenues will exhibit an increase over the next year. Instead, optimism is not significantly associated with the likelihood that the business will be open in September 2020.

**Table 4:** Optimism and business expectations.

Dependent variable:	Recovery time	Revenue increase	Business open
	(1)	(2)	(3)
Dispositional optimism	−0.0948	0.0074	0.0020
	(0.010)	(0.001)	(0.162)
Revenue change during Covid-19	−0.0323	−0.0009	0.0004
	(0.000)	(0.000)	(0.0001)
Advanced education	0.8630	−0.0223	0.0098
	(0.075)	(0.442)	(0.569)
Female	−0.6266	0.0145	0.0126
	(0.137)	(0.546)	(0.405)
Age	0.0701	−0.0023	0.0009
	(0.000)	(0.030)	(0.157)
Risk aversion	0.0067	−0.0001	0.0016
	(0.928)	(0.972)	(0.492)
Founding year	−0.0661	0.0050	0.0006
	(0.028)	(0.003)	(0.517)
Closed during Covid-19	−0.7046	−0.0205	0.3236
	(0.089)	(0.420)	(0.000)
Employees pre Covid-19	−0.0162	0.0036	0.0008
	(0.097)	(0.000)	(0.157)
Industry dummies	Yes	Yes	Yes
Observations	1,181	1,632	1,632

Note: The dependent variable is: the expected number of months to go back to pre Covid-19 revenues of December 2019 (only for those that declared a decline in revenues during the spring lockdown) in Column (1); whether or not the revenues are expected to increase by the end of 2020 as compared to those during January-April 2020 in Column (2); and the entrepreneur’s expectation about the business to be open (zero otherwise) in September 2020 in Column (3). The main explanatory variable is the continuous measure of dispositional optimism. The regressions also include a host of individual and firm-level controls, and industry dummies (coefficients not reported for brevity). p-values are reported in parenthesis.

We move to entrepreneurial expectations regarding the spread of the pandemic in the UK. Our survey contains a question where participants are asked to provide an estimate of the number of Covid-19 contagion cases at the end of July. To do so, they can choose between a set of pre-formulated answers displayed in terms of 5 thousand increments relative to the contagion cases at the time of the survey. Panel A of Table 5 shows the responses obtained (in which each subsequent category indicates a more severe diffusion of the pandemic). In Panel B of the table, we then show the results of an ordered logit regression in which the dependent variable is given by the different ordered responses shown in Panel A. Optimism appears to be negatively associated with the estimated severity of the pandemic in the UK.

**Table 5:** Optimism and expectations about the diffusion of Covid-19.

Panel A. Estimated Covid-19 cases at the end of July				Panel B. Dependent variable: Answer in Panel A	
Answer	Freq.	Perc.	Cumul.	Dispositional optimism	−0.0321
1: 274,762–279,762	108	6.6	6.6		(0.001)
2: 279,763–289,763	220	13.5	20.1	Revenue change during Covid-19	−0.0013
3: 289,764–309,764	441	27	47.1		(0.108)
4: 309,765–350,765	449	27.5	74.6	Advanced education	0.5327
5: More than 350,766	414	25.4	100		(0.000)
				Female	−0.2732
					(0.007)
				Age	0.0055
					(0.201)
				Risk aversion	0.0490
					(0.001)
				Founding year	−0.0004
					(0.940)
				Closed during Covid-19	0.0738
					(0.488)
				Employees pre Covid-19	−0.0015
					(0.648)
				Industry dummies	Yes
				Observations	1,632

Note: The left panel of the table shows the distribution of the answers to the following question: “There have been 274,762 total cases of Covid-19 in UK as of today. How many cases do you think there will be by July 31^st^?” The right panel shows the results of an ordered logit regression in which the dependent variable is represented by the answers illustrated in the left panel. The main explanatory variable is the continuous measure of dispositional optimism. The regressions include a host of individual and firm-level controls, and industry dummies (coefficients not reported for brevity). p-values are reported in parenthesis.

**Fig 4:**
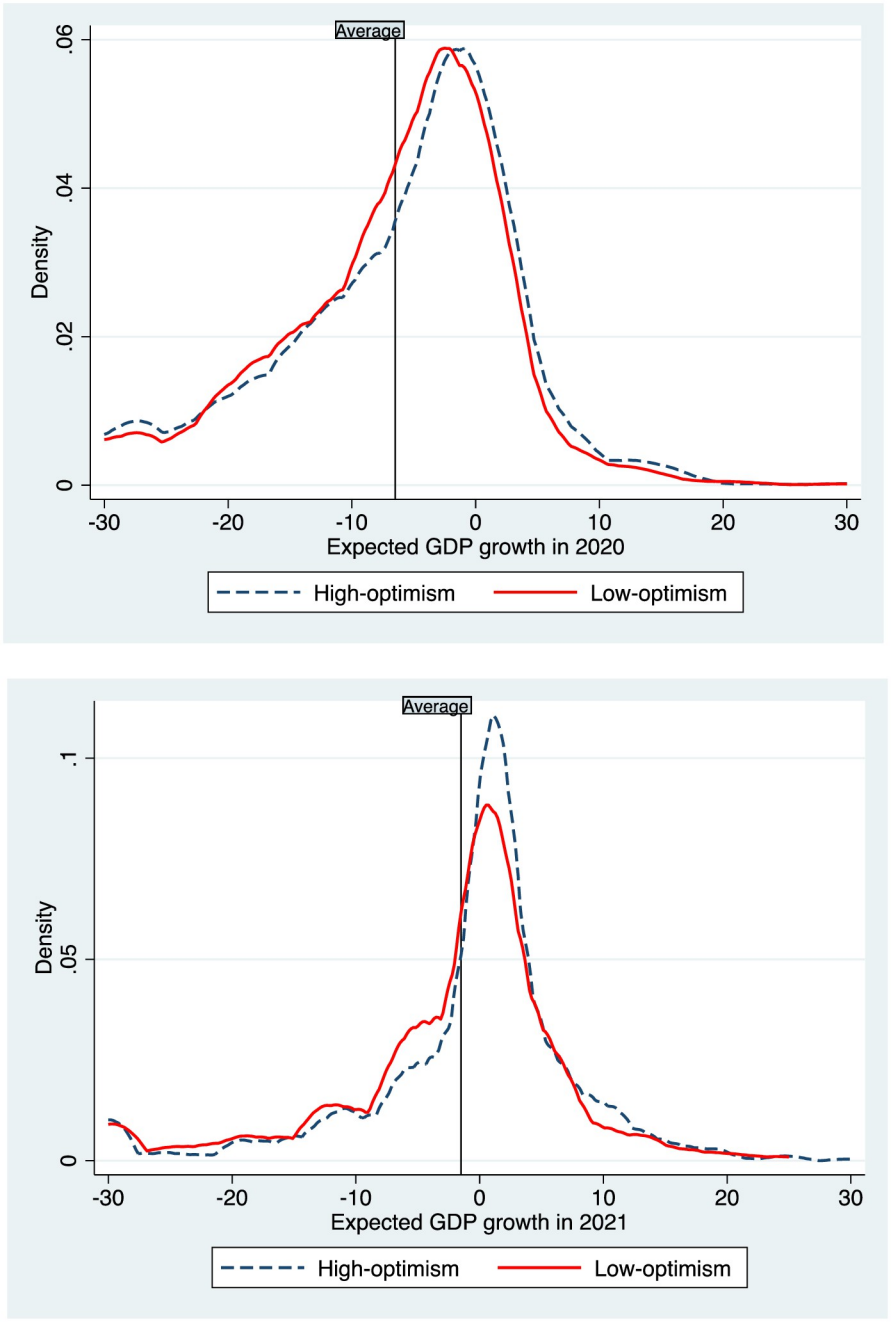
Optimism and macroeconomic expectations. The upper (lower) graph illustrates the kernel density of the responses to a question which asks entrepreneurs to estimate the GDP growth in the UK in 2020 (2021). The histograms are reported separately for entrepreneurs below and above the median threshold of optimism. The vertical lines denote the average value.

In conclusion, we estimate the association between entrepreneurial optimism and macroeconomic expectations. To do so, we take advantage of two survey questions which ask participants to estimate the UK’s GDP growth in the full years of 2020 and 2021. As Fig 4 shows, the average answers are -9% and -2%, respectively. However, consistent with our previous results, the distribution of responses from high-optimism individuals is slightly shifted to the right (i.e. they provide higher estimates of GDP growth).

This finding is confirmed by the regression analysis in Table 6, which shows how a standard deviation increase in dispositional optimism is associated with a half percentage point higher expectation of GDP growth in 2020, and a 0.8 percentage point higher expectation of GDP growth in 2021.

**Table 6:** Optimism and macroeconomic expectations.

Dependent variable:	Expected GDP growth in 2020	Expected GDP growth in 2021
	(1)	(2)
Dispositional optimism	0.0993	0.1701
	(0.036)	(0.000)
Revenue change during Covid-19	0.0048	−0.0058
	(0.244)	(0.161)
Advanced education	−0.3673	−0.5032
	(0.522)	(0.383)
Female	0.2928	−1.2871
	(0.550)	(0.008)
Age	−0.0845	−0.0497
	(0.000)	(0.029)
Risk aversion	−0.1752	−0.0487
	(0.028)	(0.553)
Founding year	0.0391	0.0443
	(0.303)	(0.233)
Closed during Covid-19	1.0758	0.4706
	(0.050)	(0.389)
Employees pre Covid-19	0.0503	0.0537
	(0.050)	(0.027)
Industry dummies	Yes	Yes
Observations	1,632	1,632

Note: The dependent variable is the entrepreneur’s expectation about: the GDP growth in the UK in 2020 in Column (1); and the GDP growth in the UK in 2021 in Column (2). The main explanatory variable is the continuous measure of dispositional optimism. The regressions also include a host of individual and firm-level controls, and industry dummies (coefficients not reported for brevity). p-values are reported in parenthesis.

### Performance

Our results so far have shown that high-optimism entrepreneurs engaged in a different set of actions (more oriented toward innovation and change) and exhibited a different set of expectations concerning the post-pandemic period. Yet, the analysis is silent about whether these attributes had a positive effect on ventures’ performance. The literature is replete with evidence showing that optimistic entrepreneurs destroy firm value due to unrealistic expectations or a tendency to discard external signals. At the same time, optimism may facilitate proactiveness and change, and hence improve the adaptation to difficult circumstances. As discussed in the data section, our follow-up survey in May 2021 contains a set of questions which allow us to measure revenue growth from 2019 to 2020, which can be used as a proxy of ventures’ success. Table 7, Column 1, shows that dispositional optimism (measured in June 2020) is positively associated with revenue growth in the full year of 2020. This result is useful to discern the value implications of the results documented so far: optimism entrepreneurs were more proactive in making changes and *also* grew more during the pandemic. Column 2 shows that the result is robust to using the measure of optimism from the second survey wave (i.e. as of May 2021).

**Table 7:** Optimism and business results.

Dependent variable: Revenue growth _2020–2019_		
	(1)	(2)
Dispositional optimism _June 2020_	1.0270	
	(0.024)	
Dispositional optimism _May 2021_		0.8016
		(0.035)
Advanced education	6.8641	7.0678
	(0.176)	(0.168)
Female	-3.9967	-3.2259
	(0.258)	(0.367)
Age	-0.3917	-0.3560
	(0.006)	(0.011)
Risk aversion	0.1950	0.0847
	(0.662)	(0.851)
Founding year	0.1068	0.0974
	(0.461)	(0.504)
Closed during Covid-19	39.8863	39.7506
	(0.000)	(0.000)
Employees pre Covid-19	0.2258	0.2230
	(0.001)	(0.001)
Industry dummies	Yes	Yes
Observations	996	985

Note: The dependent variable is the venture’s revenue growth (from 2019 to 2020). In Column (1), the key explanatory variable is dispositional optimism from the first survey wave, whereas in Column (2) is the dispositional optimism from the second survey wave. The regressions also include a host of individual and firm-level controls, and industry dummies (coefficients not reported for brevity). p-values are reported in parenthesis.

## Discussion and conclusion

While new ventures are crucial to spur technological progress and growth, they are notoriously subject to high failure rates. An important stream of research has aimed at understanding how entrepreneurs’ personal traits can help explain which businesses will thrive and which others will fail [40, 41]. Dispositional optimism is one such trait. Indeed, existing works in this area show that optimism has far-reaching implications not only for individual behaviors [36] but also for the businesses that individuals lead. Some studies have found that optimistic entrepreneurs invest in disparate innovative projects [14] and tend to experience lower financial performance [15], while others have shown that optimism is conducive to organizational change [23] and is positively associated with business success [42]. Our contribution to this literature has been to explore the relationship between optimism and entrepreneurial actions during the Covid-19 pandemic, which drove out of business a significant number of firms and prompted a massive reallocation of output across and within industries. Understanding the ability of entrepreneurs to face these unprecedented challenges is thus crucial for both managerial and policy-making perspectives.

Our evidence, based on a comprehensive survey on UK entrepreneurs, indicates that new ventures experienced significant damages during the spring outbreak of Covid-19, though the impact was highly heterogeneous across firms. The key finding emerging from our study is that optimism helps to explain part of this heterogeneity across firms. In particular, we find that firms led by optimistic entrepreneurs had a higher likelihood of innovation and organizational changes, which were useful to weather the pandemic shock. Moreover, optimistic entrepreneurs display more positive beliefs toward future events: they expected their businesses to fully recover over shorter periods of time and, more generally, they had rosier expectations about macroeconomic conditions. Finally, examining data on ventures’ performance, our data reveal that high-optimism entrepreneurs grew more than low-pessimism entrepreneurs over the entire pandemic year of 2020. Collectively, our study reveals a bright side of dispositional optimism: it improves entrepreneurs’ readiness and facilitates successful adaptation to a negative shock.

Before concluding, we shall acknowledge that—despite the inclusion of a comprehensive set of control variables in the regression analysis—our study remains correlational in nature. To tease out more directly the causal effect of optimism on the ability of individuals to overcome a shock, a fruitful approach could be to leverage manipulations of optimism in a laboratory setting. Alternatively, future studies could use cross-sectional variations in optimism while exploiting the periods before and after a temporally well-defined shock (unlike Covid-19, which does not have a sharp ending point and thus does not permit a clear comparison of pre/post-shock periods).

## Supporting information

S1 File Table A1. Classification of sectors. Table A2. Estimated recovery period—by optimism level.
(DOCX)
