# Supplementary material for: Dispositional optimism and business recovery during a pandemic
Source: PLoS One. 2022 Jun 9;17(6):e0269707. doi: 10.1371/journal.pone.0269707 (PMC9182296; doi:10.1371/journal.pone.0269707)
Supplement: S1 File — Table A1. Classification of sectors. Table A2. Estimated recovery period—by optimism level. (DOCX) [file pone.0269707.s001.docx]

**S1 File**

**Classification of sectors**

| Accommodation and Food Services |
| --- |
| Administrative and Support and Waste Management and Remediation Services |
| Agriculture, Forestry, Fishing and Hunting |
| Construction |
| Educational Services |
| Finance and Insurance |
| Health Care and Social Assistance |
| Information |
| Management of Companies and Enterprises |
| Manufacturing |
| Mining, Quarrying, Oil and Gas Extraction |
| Other Sector |
| Professional, Scientific, and Technical Services |
| Real Estate, Rental and Leasing |
| Transportation and Warehousing |
| Utilities |

**Estimated recovery period - by optimism level**

|  | High-optimism | | | Low-optimism | | |
| --- | --- | --- | --- | --- | --- | --- |
| Estimated recovery period: | Obs. | Freq. | Cumul. | Obs. | Freq. | Cumul. |
| Within 6 months | 325 | 56.92 | 56.92 | 314 | 51.48 | 51.48 |
| Within 12 months | 180 | 31.52 | 88.44 | 229 | 37.54 | 89.02 |
| Within 18 months | 29 | 5.08 | 93.52 | 29 | 4.75 | 93.77 |
| Within 24 months | 28 | 4.90 | 98.42 | 31 | 5.08 | 98.85 |
| More than 24 months | 9 | 1.58 | 100.00 | 7 | 1.15 | 100.00 |

Note: The columns “High-optimism” and “Low-optimism” denote entrepreneurs above or below the median threshold of optimism.
